# Validation of a Light EEG-Based Measure for Real-Time Stress Monitoring during Realistic Driving

**DOI:** 10.3390/brainsci12030304

**Published:** 2022-02-24

**Authors:** Nicolina Sciaraffa, Gianluca Di Flumeri, Daniele Germano, Andrea Giorgi, Antonio Di Florio, Gianluca Borghini, Alessia Vozzi, Vincenzo Ronca, Rodrigo Varga, Marteyn van Gasteren, Fabio Babiloni, Pietro Aricò

**Affiliations:** 1 BrainSigns Srl, Lungotevere Michelangelo 9, 00192 Rome, Italy; gianluca.diflumeri@uniroma1.it (G.D.F.); danielegermano92@gmail.com (D.G.); andrea.giorgi@brainsigns.com (A.G.); antonello.diflorio@brainsigns.com (A.D.F.); gianluca.borghini@uniroma1.it (G.B.); alessia.vozzi@uniroma1.it (A.V.); vincenzo.ronca@uniroma1.it (V.R.); fabio.babiloni@uniroma1.it (F.B.); pietro.arico@uniroma1.it (P.A.); 2 Department of Molecular Medicine, Sapienza University of Rome, Piazzale Aldo Moro 5, 00185 Rome, Italy; 3 ITCL Technology Centre, C. López Bravo, 70, 09001 Burgos, Spain; rodrigo.varga@itcl.es (R.V.); marteyn.vangasteren@itcl.es (M.v.G.); 4 College of Computer Science and Technology, Hangzhou Dianzi University, Hangzhou 310005, China

**Keywords:** stress, EEG, driving, random forest, wet EEG sensors

## Abstract

Driver’s stress affects decision-making and the probability of risk occurrence, and it is therefore a key factor in road safety. This suggests the need for continuous stress monitoring. This work aims at validating a stress neurophysiological measure—a Neurometric—for out-of-the-lab use obtained from lightweight EEG relying on two wet sensors, in real-time, and without calibration. The Neurometric was tested during a multitasking experiment and validated with a realistic driving simulator. Twenty subjects participated in the experiment, and the resulting stress Neurometric was compared with the Random Forest (RF) model, calibrated by using EEG features and both intra-subject and cross-task approaches. The Neurometric was also compared with a measure based on skin conductance level (SCL), representing one of the physiological parameters investigated in the literature mostly correlated with stress variations. We found that during both multitasking and realistic driving experiments, the Neurometric was able to discriminate between low and high levels of stress with an average Area Under Curve (AUC) value higher than 0.9. Furthermore, the stress Neurometric showed higher AUC and stability than both the SCL measure and the RF calibrated with a cross-task approach. In conclusion, the Neurometric proposed in this work proved to be suitable for out-of-the-lab monitoring of stress levels.

## 1. Introduction

There are several different definitions and kinds of stress. Stress can be mental or physical, acute or chronic, but in substance, it is a very personal matter. In all these cases, in fact, underpinning the stress response is how a subject can manage the demand of the environment in relation to his or her capacity to make a decision and cope with circumstantial stressors [1]. 

During driving, it is easy to meet stressful factors. Typical stressors during driving tasks were classified into five categories: difficult driving due to the road, social interaction, unexpected events, delay, and other drivers’/pedestrians’ behaviour [2]. All these factors increase stress levels due to the perception of a higher risk of error and uncertainty [3]. Different research has demonstrated the effects of traffic and road conditions on driver-perceived stress both in simulated [4] and real highway and city courses [5]. In addition to the aforementioned external factors, there are internal factors, such as the driver’s mental workload, that can also contribute to increases in stress level [6,7]. According to the psychological model for driving automation described in the Ref. [8], there is a bidirectional relationship between workload and stress: stress is affected by workload level, moreover, the effort involved in coping with stress actually adds to the task demands. Finally, the stressful answer is strictly connected to the expectation of a threat, due to memory and species-specific inclination, like negative judgments in humans [9].

Both task-related and environmental stressors impact cognitive processing, which in turn induce an impairment of performance [3]. This causality is usually explained by leveraging on the arousal construct. Arousal was associated with performance, as demonstrated by the well-known inverted-U function of the Yerkes–Dodson law [10]. Excessive arousal can affect cognitive processes at the basis of decision-making, attention, and memory, inducing a performance decrement [11]. 

Therefore, stress is a key factor for road safety and for Ergonomics, since it affects the driver’s decision-making and risk assessment ability. A fundamental question in this context is how to measure stress in order to be able to predict when the subject’s coping behaviour is about to fail. For this, a reliable and continuous measure of stress levels during driving is necessary. This is one of the main aims of Neuroergonomics, the science concerned with the study of the brain in relation to performance in everyday settings [12]. Leveraging the neurophysiological measures and human cognition, the Neuroergonomics approach allows to overcome the limitations of subjective and discrete measures of driver mental states [13]. In fact, the main accepted way to obtain a subjective perception of the stress level is by using questionnaires [14]. Nevertheless, questionnaires can only give an overview of what the subject’s general feeling of stress is, but not the change of stress over time, and, as subjective measures, these may not be reliable, but affected by perception, mood, and character. 

In the driving context, neurophysiological signals are a useful metric for providing feedback about a subject’s state. Compared to questionnaires’ weaknesses, neurophysiological measures can be used to assess the driver’s state because they can be collected continuously and without interfering with the driving [13,15]. This is possible because the body answers to stress-secreting hormones, such as adrenaline. In turn, this causes physical changes, such as increased heart rate, respiratory rate, blood pressure, muscle tension, skin sweating and pupil dilation [16]. These physiological responses are widely used for stress assessment. 

In particular, skin sweating is positively correlated with variations of the Skin Conductance (SC) signal, that becomes an indicator for sympathetic activation due to the stress reaction [17]. Sweating gland activity is mainly controlled by the sympathetic nervous system (SNS), leading to increasing skin electrical conductivity during stressful events. Because of the relation between skin sweating and the consequent electrical properties of the skin, the measurable physiological phenomenon is known as ElectroDermal Activity (EDA) [18]. The most important features computed from EDA are: (i) Skin conductance level (SCL), the slowly varying component (also known as Tonic component) of the EDA signal, mainly related to the overall psychophysiological activation and arousal of the user; (ii) skin conductance response (SCR), the fast varying component (also known as Phasic component) of the EDA signal, including the event-specific reactions of the human palmar/plantar sudomotor system as the somatosympathetic reflex (thus not due to the thermoregulatory function) [19]. Both SCL, in terms of average level, and SCR, in terms of frequency, amplitude, and latency of response peaks, increase when a subject is under stress [20,21]. In the particular field of car driving, according to the Ref. [5] that conducted an experiment on 24 subjects during real driving, SCL was considered the most effective feature correlating with stress. 

Measuring physiological signals in ecological environments, such as during driving activity, is more difficult than in a laboratory environment. For the controlled experiments in the laboratory, different tasks and stressors were validated, as they make it possible to reach the opportune level of stress: the Trier Social Stress Test [22], Cold Pressor test [23], Montreal Imaging stress task [24], and the Stroop colour word interference test [25]. Moreover, the combination of some of these in multitasking batteries like in the Ref. [26] has often been used to elicit stress. In fact, when working memory is highly stressed because of higher task demand, there is a deterioration of inhibition and shifting functions (control executive functions), shown in the worsening of performance related to selective and divided attention [11]. In contrast, during real activities, multiple stressors occur, making it difficult to establish defined stress levels to label recorded data [27]. Moreover, signal artifacts caused by movement could affect the accuracy of measured recordings, and the physiological responses caused by mental stress can be masked by variations due to physical activity itself [28]. 

To overcome such limitations, several studies have moved to EEG stress assessment thanks to the possibility to directly access central nervous system (CNS) activity. In fact, CNS activates both the hypothalamic-pituitary-adrenal (HPA) axis and the autonomic nervous system (ANS), in particular, the sympathetic adrenomedullary system (SAM). Respectively, these two events happen more often when there is a menace of social evaluation (this produces the HPA axis response seen when salivary cortisol arises) and due to cognitive or physical effort (that induced the SAM response showed in HR and salivary alpha-amylase) [29]. Thanks to fMRI, it was possible to recognize the key brain areas in stress response: Amygdala, Hippocampus, Superior Frontal gyrus, Inferior Frontal gyrus, Inferior Parietal gyrus, Superior Parietal gyrus and Superior Temporal gyrus [30]. However, in realistic applications, it is not possible to have access to such brain activity both for the intrusiveness of fMRI methods and for its low temporal resolution. On the other hand, the high temporal resolution and the possibility to use the EEG as an unobtrusive neuroimaging method make it one of the most reliable stress measurements. From this point of view, it was assessed that in the presence of stressors, there is a decrease in prefrontal cortex activity in alpha power and an increase in beta in temporal and parietal sites [31,32]. The beta band has a significant positive interaction with stress and is correlated with cortisol levels [30,33,34]. So far, several attempts were made to also define a real-time measure of stress based on EEG signals using machine learning methods [34]. Machine learning (ML) is the union of mathematical algorithms and models that, applied to a certain amount of data, allows to perform classification, regression, or features selections. So far, all these machine learning approaches were effectively employed with EEG features in both clinical and non-clinical domains [35,36]. In particular, in the Neuroergonomics field, several studies have used ML techniques to classify human mental states [13,37]. The most commonly used algorithms are support vector machines (SVM), k-nearest-neighbours (KNN), decision trees (DT), and Random Forest (RF). Each algorithm has pros and cons and could perform differently in different applications. In this work, we chose the RF because, being an ensemble method, it allows for good generalization to new data [38] and is more robust to overfitting than the individual tree [39]. Moreover, the RF is a model that is easy to explain: thanks to the feature importance, it is possible to know how the model is affected by each feature. Finally, it is a non-linear method; therefore, it is suitable for finding potentially complex structures in the data, compared to linear models that do not work well in the presence of strong noise or outliers, if the regularization has not been done well or the problem is intrinsically non-linear [40,41], things that are very likely in the real environment. 

A recent review on stress assessment methods using EEG signals has resumed the main findings in this context, emphasizing the discrepancies mainly due to the diverse number of variables, brain regions of interest, type of stressor, duration of experiment, EEG signal processing, feature extraction technique, and the type of classifiers employed [34]. It highlighted the necessity to develop an online system that deals with stress recognition in real-time and that is able to solve the calibration issues. As said, the labelling of different levels of stress, and/or the identification of stressed/unstressed groups, remains one of the open issues in stress assessment in the real environment.

This work aims to validate a light EEG-based measure consisting of just two channels and wet-based sensors for monitoring stress levels in real-time and without any calibration during a realistic driving task. The stressful condition was elicited using both task-related and external stressors like traffic, time pressure, noisy environment and social evaluation. The proposed Neurometric was firstly tested during a laboratory multitasking task. A modified version of the defined intensity stressor simulation (DISS) [26] was implemented to elicit low and high levels of stress modulating task difficulty, time pressure, and negative feedback.

## 2. Materials and Methods

### 2.1. Experimental Setup

Twenty healthy subjects (29.3 ± 5.12 years old) took part in the study—eight females, and twelve males—recruited on a voluntary basis. As inclusion criteria, a driver’s licence was required, as well as the ability to drive a car with a manual transmission. The experiments were conducted following the principles outlined in the Declaration of Helsinki of 1975, as revised in 2000. Experiments were approved by the Ethical Committee of Sapienza University. Informed consent was obtained from each subject on paper, after the study explanation, and all the data were pseudorandomized to prevent any association with the subject’s identity.

For this experiment, a standard EEG cap with water-based electrodes (saltwater sponges and passive Ag/AgCl electrodes) and LiveAmp amplifier (Brain Products GmbH, Gilching, Germany) was used. These electrodes allow the recording of high-quality EEG signals, with no gel residues. The EEG signals were recorded with a sampling frequency of 250 Hz, referenced to earlobes, and the investigated scalp positions were AFz, AF3, AF4, AF7, AF8, Pz, P3, and P4. Water-based electrode contact was considered good, for impedance values below 100 KOhm [42]. 

Moreover, the ElectroDermal Activity (EDA) was recorded synchronously with the EEG signal through the Shimmer3 device (Shimmer Sensing Ltd., Dublin, Ireland) positioned on the non-dominant hand. In particular, the two electrodes were positioned on the first phalanx of the index and middle fingers, respectively, and the recordings had a sampling frequency of 64 Hz. 

After the subject preparation, the experiment started, and each subject was asked to perform the multitasking and the driving task. The day before the experiment, all the subjects were trained to handle both tasks to avoid any learning effect. 

### 2.2. Multitasking

**Figure 1:**
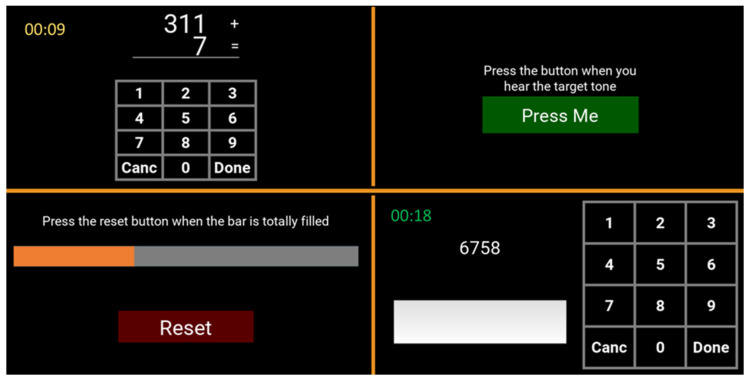
Multitasking application screen. In clockwise order: the auditory monitoring, the phone number entry task, the visual monitoring and the mental arithmetic task.

The implemented multitasking application was a modified version of the defined intensity stressor simulation (DISS) already used to increase self-ratings of negative mood, arousal and stress-related physiological responses [26]. The multitasking app comprises a set of four concurrent cognitive tasks of varying difficulty, presented via a split screen (Figure 1). The four chosen tasks are:

1. Mental Arithmetic (Left-Up): addition results must be entered into the numeric touch screen keypad. As the difficulty increases, the number of digits (from 1 to 3) and carryover digits (from 0 to 2) increase.
2. Auditory Monitoring (Right-Up): A target tone must be identified between two tones of different frequencies emitted at regular intervals. As the difficulty increases, the target tone and distractor tone increase in similarity.
3. Visual Monitoring (Left-Down): A horizontal fill bar should be reset as soon as it becomes full. As the difficulty increases, the fill rate increases.
4. Phone Number Entry Task (Right-Down): A number must be entered on the touch screen keypad. As the difficulty increases, the number of digits to be entered increases (from 4 to 10).

First, subjects were asked to perform the four tasks individually (to refresh their memory on how to handle each specific task). In this case, a 30 s condition was performed for the Auditory monitoring, Visual Monitoring and the Phone Number Entry Task, whereas two separate 30 s conditions (at a different level of difficulty) were established for the mental arithmetic task. After that, subjects performed the four concurrent tasks (multitasking phase), in particular, seven levels of 1 min multitasking at increasing difficulty. According to the literature, performing several concurrent tasks compared to the single-task approach induces an increase in mental workload [26,43]. In addition, increased difficulty induces a greater perception of time pressure and frustration, resulting in increased stress. The first two levels were considered as low-stress levels, and the last two as high-stress levels. 

Performance measures were collected during each multitasking level. For each subtask, an ad hoc index of performances was computed with values in the range of 0–100. 

For the Mental Arithmetic task, the performances were computed as:

$$100\times \frac{num\;of\;right\;additions}{max\;num\;of\;right\;additions}$$

where the *max num of additions* is the maximum number of additions performed by the subject during the seven levels, and *num of right additions* is the number of right answers referred to the specific level. 

For the Auditory Monitoring task, the performances were computed by averaging the quantity for each answer:

$$100\times \frac{5-ReactionTime}{5}$$

where *ReactionTime* is the interval between the sound and when the subject taps the “Press me” button, taking into account that five is the interval between two stimuli. In the case of wrong answers (the subject pressed the button to non-target stimulus), the *ReactionTime* is considered equal to 5.

For the Visual Monitoring, the performances were computed by averaging the quantity for each answer:

$$100-\left(Bar2Fill+100\times \frac{ReactionTime}{Time2Fill}\right)$$

where *Bar2Fill* is the percentage of the bar to completion; *ReactionTime* is the interval between bar completion and when the subject taps the “Reset” button; and *Time2Fill* is the time needed to have the bar completely full (it is used to convert the reaction time to “equivalent in bar” according to the proportion *Time2Fill: 100 = ReactionTime: BarEquivalent*).

For the Phone Number Entry Task, the performances were computed as:

$$100\times \frac{num\;of\;right\;entries}{max\;num\;of\;entries}$$

where the *max num of entries* is the maximum number of entries provided by the subject during the seven levels, and *num of right entries* is the number of right answers referred to the specific level. 

An overall performance index was obtained by averaging the previous four indexes and expressing it in percentage terms. 

### 2.3. Realistic Driving 

**Figure 2:**
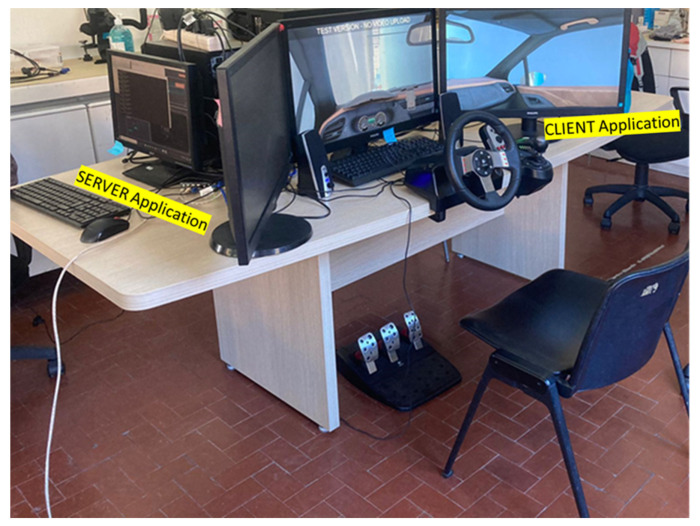
Car simulator. The SERVER and CLIENT applications have been highlighted.

The car simulator is a typical real car setup (seat and dashboard with steering wheel, gearshift and all the common commands), while the virtual environment is reproduced through three screens (Figure 2). The software of the car simulator employed for the experiment was provided by the ITCL Technology Centre (Spain) for the purpose of the Mindtooth Project (GA 950998) in which this experiment was contextualized. Here, instead of the full car cockpit available with the commercial simulator, a light version of it was readapted and used, with a commercial steering wheel (Logitech G27) and three 27” screens. This configuration was enough to make the driving environment immersive for the participants, thanks to the high realism of the driving environment. There were two computers, one running a “SERVER Application”, to start and stop each specific run, and another one running a “CLIENT Application” to reproduce the driving environment. The subjects were asked to follow the navigator instruction shown on the central screen and to perform two exercises: the first exercise was set in a circuit, the second in a city with simulated traffic. To elicit stress, subjects were instructed to perform these two-run repetitions with three stressors:

1. Time pressure: subjects have a limited time to make the same route. A chronometer was used to show the remaining time.
2. White Coat effect: An operator was just behind the driver, taking notes of errors committed.
3. External noise: Car traffic noise was introduced during the exercise.

The performances of each repetition were recorded through the simulator itself. The car simulator generates a log file with all the specific events that happened within each scenario execution. Among the possible available events, collisions of the car with anything in the simulated worlds were considered as a performance indicator. 

### 2.4. Subjective Assessment: NASA-TLX Questionnaire

At the end of each run, experimental subjects were asked to fill out an electronic version of the NASA—Task Load Index (NASA-TLX) questionnaire [44,45]. The questionnaire was organized in six different dimensions: mental demand, physical demand, temporal demand, performance, effort, and frustration. The unweighted version of the questionnaire was used, and therefore, a score from 0 to 100 was obtained for each analyzed subscale.

### 2.5. Physiological Assessment: Electrodermal Activity

The EDA is the signal related to Skin Conductance (SC). An increase in skin conductance level (SCL) is considered a reliable indicator of human arousal variations [46], and it is typically used to evaluate the anxiety produced by a situation or the stress related to a threat [47,48,49]. The EDA was preprocessed through a MATLAB pipeline: it was downsampled from 64 to 8 Hz and then filtered through a fifth-order Butterworth low-pass filter, with the cut-off frequency at 2 Hz, to remove all the higher-frequency components that are not related to the electrodermal activity, such as artefacts due to movements and fast, high pressure in electrode–skin contact. In particular, the latter causes fast and short signal peaks over the EDA signal: in this case, a double-step procedure (automatic detection and expert visual check) was implemented in order to recognize the artefactual transient and to correct it by interpolating the corresponding signal through a piecewise cubic spline [50]. Then, the signal was processed by using the Ledalab suite, a specific open-source toolbox implemented within MATLAB for the EDA processing; the Continuous Decomposition Analysis [51] was applied to separate the SCL and the SCR components of the EDA. 

The mean value of the SCL during the experimental conditions was used in this study as a physiological correlate of stress.

### 2.6. Neurophysiological Assessment: Electroencephalographic Signals

The EEG signal was firstly band-pass filtered with a fifth-order Butterworth filter in the interval 2–30 Hz. 

The blink artefacts were detected by means of the Reblinca method [52] and corrected by leveraging the ocular component estimated through a multi-channel Wiener Filter (MWF) [53]. EEG signals were segmented into epochs of 1 s, and if the EEG signal amplitude exceeded ±80 (μV), it was marked as an artefact (threshold criterion). After the analysis of artifacts, two participants were removed.

From the artefact-free EEG, the Global Field Power (GFP) was calculated for the EEG frequency band of interest, which was Beta High from 21 to 26 Hz. This band was defined accordingly with the Individual Alpha Frequency (IAF) value [54]. Since the alpha peak is mainly prominent during rest conditions, the subjects were asked to keep their eyes closed for a minute before starting the experiment. Such a condition was then used to estimate the IAF value specifically for each subject. Consequently, the EEG Beta High band was defined as Beta High = (IAF + 11): (IAF + 16) Hz. 

The stress Neurometric was defined according to the literature, and previous results as:

Neurometric = Beta High(P3, P4)

that is, the average of the brain activity in the high-beta band over the parietal brain area. 

### 2.7. Machine Learning Model

GFP values computed in the Beta High band over the P3 and P4 electrodes were used as features to train a Random Forest (RF) model for stress level classification. The Random Forest was chosen because of its good balance between the level of reliability and execution time [39]. Following preliminary analysis, it was decided to optimize the parameters that most influence the results:

o Number of estimators: range from 50 to 500;
o Max depth: range from 2 to 30;
o Min samples split range from 0.01 to 0.5;
o Min samples leaf range from 0.01 to 0.5; and
o Max leaf nodes range from 2 to 40.

Each observation belonging to a specific run was labelled according to the level of stress expected for that run. In the case where the classes (e.g., high-stress, low-stress) were too unbalanced because of the number of rejected artefacts, the Adaptive Synthetic (ADASYN) method was used to provide synthetic resampling [55]. This algorithm allows to generate synthetic data to oversample the minority class.

Once the observations were balanced for each class, the classifier was trained following two different approaches: an intra-subject and a cross-task approach. 

In the intra-subject classification, a model was created for each subject individually and limited to the multitasking and the driving task: for each of the two tasks, both training and testing was performed using the observations coming from the same subject. One repetition was used to train the algorithm and optimize the parameters, and one to test the algorithm. This was performed for all the possible combinations, so that all sessions were involved in both the training and testing phases of the algorithm, in order to minimize the risk of overfitting and obtain a global estimate of the classifier capabilities.

The cross-task approach differs from the intra-subject because the training phase employed a set of data coming from multitasking and the testing phase employed the data recorded during the driving task. 

### 2.8. Evaluation of Models Effectiveness

To evaluate the effectiveness of each model, the Area Under Curve (AUC) of the Receiver Operating Characteristic (ROC) [56] was computed and averaged in each session. We decided to use this metric since it is independent of any discrimination threshold, and so it could also be applied to the SCL and the Neurometric, to have a balanced comparison among all the measures. In particular, for each data point of data testing, the probability of belonging to the high-stress class was calculated as an output from the RF model, both for the intra-subject and cross-task approach. Instead, for the Neurometric and SCL evaluation, the mean value computed on the same testing dataset was used. A moving average on these values was then applied, to simulate different time resolutions (i.e., decimations), starting from 1 up to 60 s (maximum value to be able to evaluate a reliable AUC value) by 10 s steps.

### 2.9. Statistical Analysis

Non-parametric statistical tests were performed, since the obtained distributions were not normally distributed. Following this, the Friedman test (α = 0.05) was used to compare the AUC values obtained through the stress Neurometrics with the intra-subject, the cross-task, and the SCL models, respectively. The Dunn test was performed for post hoc analysis. To compare the subjective and performance measures, the Wilcoxon signed-rank tests (α = 0.05) were performed to analyze the differences between the high-stress and the low-stress conditions.

Finally, the continuous measures of SCL and the Neurometric were compared with the score curve of the classifier: each identified model was used to predict the probability of a specific observation to belong to the high-stress class. These curves were also compared in terms of Pearson correlation and distances between the curves using the Root mean squared error (RMSE). 

## 3. Results

In the following, we present the results regarding the multitasking and the driving experiments in terms of behavioural and subjective measurement, AUC value comparison, and temporal analysis at different decimations. 

### 3.1. Multitasking

**Figure 3:**
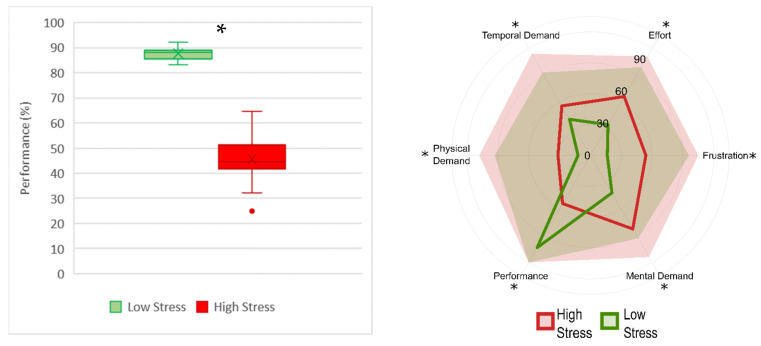
Boxplot representation of the performance in terms of percentage (**left**) and radar chart of NASA-TLX subscales (**right**). In the radar chart, the lines represent the median of each subscale and the shaded areas represent the maximum value in the population. The low-stress condition is represented in green, and high-stress in red. The asterisks show that *p* < 0.05 resulting from the Wilcoxon signed-rank test.

The multitasking experiment was analysed in terms of both performance and subjective measures during the two conditions (Figure 3). The subjects showed significantly higher performance (Z = 3.92, *p* < 0.0001) during the low-stress condition. Moreover, the subject perceived a higher level of frustration (Z = −3.83, *p* = 0.0001), temporal demand (Z = −3.77, *p* = 0.0001), physical demand (Z = −3.32, *p* = 0.0001), mental demand (Z = −3.68, *p* = 0.0002), effort (Z = −3.66, *p* < 0.001), and lower performance (Z = 3.82, *p* = 0.0001) during the high-stress condition.

**Figure 4:**
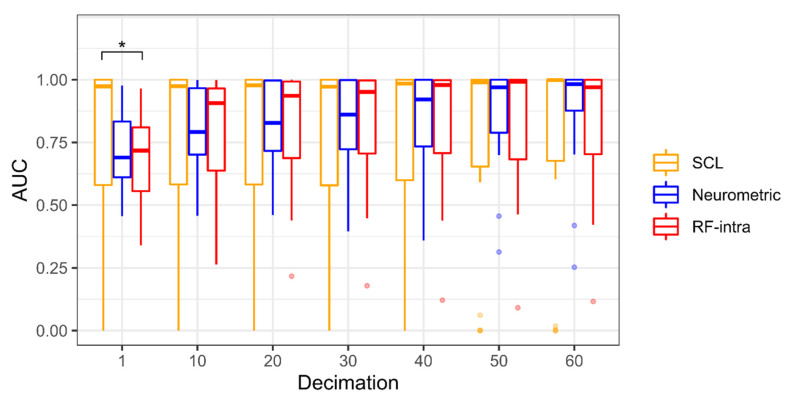
Boxplot representation of Area Under Curve (AUC) for seven different decimations (1 to 60 by 10 s step) of the multitasking experiment. The Random Forest (RF-intra) is represented in red, the Neurometric in blue, and the Skin Conductance Level (SCL) in orange. The points represent the outliers, and the asterisk shows that *p* < 0.05 resulting from the Dunn’s post-hoc tests.

Figure 4 shows the statistical comparison between the Random Forest (RF-intra), Neurometric and SCL performances in terms of AUC. As the time resolution decreases, the performance increases. The Friedman test is significant only for 1 s decimation (χ^2^(18) = 7.11, *p* = 0.03). The boxplot representation shows that the median of RF-intra and SCL is higher than those reached by the Neurometric, however, there is a statistical significance only for 1 s of decimation (Z = −2.04, *p* = 0.04): the SCL is significantly higher than RF-Intra. For all other decimations, there is no difference between the three models, however, the SCL distribution shows higher dispersion for decimation lower than 50 s compared to both the Neurometric and RF-intra, showing the high variability among subjects (Table 1). 

**Figure 5:**
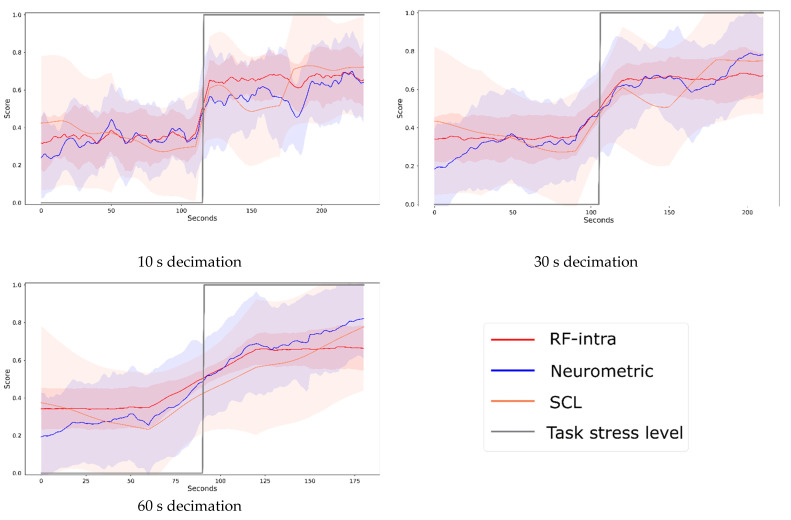
Stress during the multitasking experiment. The time series were obtained from the three different models at three different decimations (10, 30 and 60 s). The line represents the average over the population of recorded stress value, the shadow represents the standard deviation. In grey the expected value of stress level (low- and high-stress conditions) is represented, the Random Forest (RF-intra) is represented in red, the Neurometric in blue, and the Skin Conductance Level (SCL) in orange.

Figure 5 shows the trend of stress obtained through the three investigated models for three different decimations (10, 30, 60 s). The line represents the average measure of stress over the population and the shadow represents the standard deviation. For the sake of representation, all measures were normalized between 0 and 1. In Table 2, the correlation and distance between the curves are described. 

### 3.2. Realistic Driving

**Figure 6:**
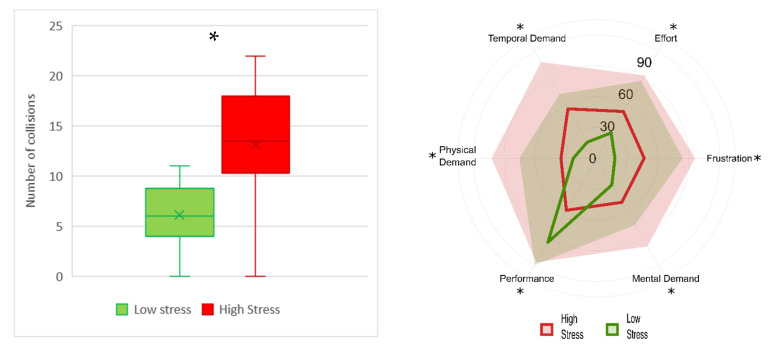
Boxplot representation of the number of collisions (**left**) and radar chart of NASA-TLX subscales (**right**). In the radar chart the lines represent the median of each subscale and the shaded areas the maximum value in the population. The low-stress condition is represented in green, and high-stress in red. The asterisks show the *p* < 0.05 resulting from the Wilcoxon signed-rank test.

The realistic driving performances were analysed in terms of number of collisions (Figure 6). During the high-stress condition, the subjects showed a significantly higher number of collisions (Z = −3.55, *p* < 0.001). Moreover, during the high-stress condition the subjects perceived significantly higher frustration (Z = −3.83, *p* = 0.0001), temporal demand (Z = −3.73, *p* < 0.001), physical demand (Z = −3.23, *p* = 0.001), mental demand (Z = −3.1, *p* < 0.01), effort (Z = −3.44, *p* < 0.001), and significantly lower performance (Z = 3.4, *p* < 0.001). 

**Figure 7:**
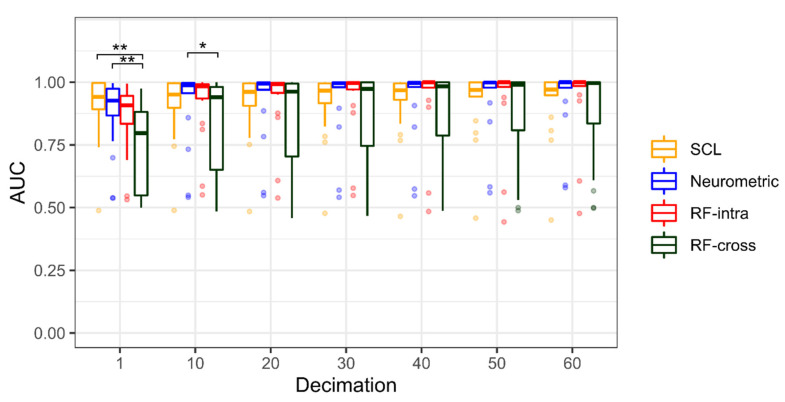
Boxplot representation of Area Under Curve (AUC) for seven different decimations (1 to 60 by 10 s step) of driving experiment. The Random Forest calibrated cross-task (RF-cross) is represented in green, the Random Forest calibrated intra-subject (RF-intra) in red, the Neurometric in blue, and the Skin Conductance Level (SCL) in orange. The points represent the outliers and the asterisk shows that *p* < 0.05 (the double-asterisk means *p* < 0.01) resulting from the Dunn’s post-hoc tests.

Figure 7 shows the statistical comparison between the RF-cross, RF-intra, Neurometric and SCL for the driving experiment. Additionally, in this case, as the time resolution decreases, the performance increases. The Friedman test is significant for each decimation (*p* < 0.001 in each case). However, the post hoc analysis shows significant differences only for 1 and 10 s decimations. RF-cross performs significantly worse than SCL (Z = −3.27, *p* = 0.001), and Neurometric (Z = −2.67, *p* < 0.01) for 1 s of decimation, and significantly worse than Neurometric (Z = −2.14, *p* = 0.03) for 10 s of decimation. In this case, the RF-cross shows the highest dispersion (Table 3).

**Figure 8:**
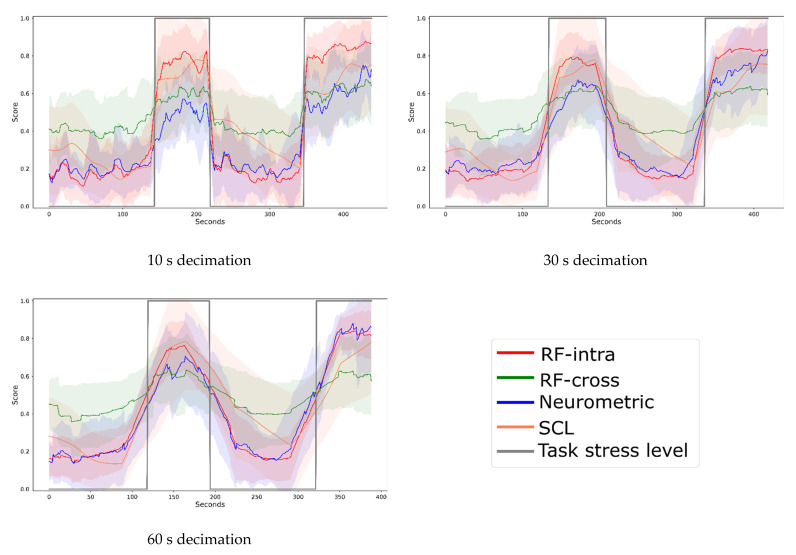
Stress during the driving experiment. The time series were obtained from the four different models at three different decimations (10, 30 and 60 s). The line represents the average over the population of recorded stress value, and the shadow represents the standard deviation. The expected value of stress level (low and high-stress condition) is represented in grey, the Random Forest calibrated cross-task (RF-cross) is represented in green, the Random Forest calibrated intra-subject (RF-intra) in red, the Neurometric in blue, and the Skin Conductance Level (SCL) in orange.

Figure 8 shows the trend of stress obtained from the four investigated models for three different decimations (10, 30, 60 s) graphically and in terms of correlation and distance between the curves (Table 4).

## 4. Discussion

This study aimed at validating an EEG-based metric for real-time stress measurement. In particular, it was tested during a multitasking session and then validated during a realistic driving task. The Neurometric was compared with other stress measurements like SCL- and machine learning-based measures calibrated using both an intra-subject and cross-task approach. 

The proposed Neurometric for continuous stress assessment consists of the average of parietal brain activity in the high-beta band. This choice was made for both theoretical and experimental reasons. On the one hand, researchers found a positive correlation between EEG beta power rhythms with stress [57], and in particular with activity in temporal and parietal sites [58,59]. Other works showed that the temporal lobes are particularly sensitive to stressors like traffic noise and, also, a positive correlation was found between mental stress and EEG activity in the beta band [59,60,61]. On the other hand, such spatial and spectral information has several advantages, as it leaves the forehead, essential for working memory and executive control, free to be used for measuring other interesting mental states, such as mental effort. This will reduce the probability of incurring confounding effects between the measure of the two different mental states also from a spectral point of view, since mental effort is usually measured in the theta band [62].

The inference of a unique human-factor construct based on unique physiological indices is still unresolved, since different interrelated states can impact on each neurophysiological measure [63]. However, in contrast to real-world settings where different human factor constructs (e.g., stress, workload, attention) usually overlap with each other, in controlled laboratory tasks, one construct can be manipulated at a time, and correspondent neurophysiological variation can be retrieved. Therefore, the proposed Neurometric of stress was firstly validated during a controlled multitasking session. The multitasking experiment can be considered a typical rigorous laboratory task, effectively employed for inducing real-world stress (e.g., multitasking activity, increasing difficulty, time pressure) to subjects, in contrast to more realistic settings [64]. During the laboratory task, the stress level was manipulated by stressors considered highly effective in literature: high temporal demand, social evaluation and a noisy environment [29]. These stressors were added to a multitasking game. A similar task already used in the literature proved that it is possible to elicit stress by increasing the number of tasks a user must attend to, or by increasing the workload intensity of the tasks [26]. Parallel experimental choices were made for the driving task in which drivers were asked to drive fast, with high traffic noise and while their performance was being judged. More than in laboratory experiments, confirmatory independent measures are important to assess the validity of the study in the case of realistic settings [65]. We can speculate that the experimental manipulation was effective in both cases thanks to behavioural and subjective evidence. During the high-stress condition of both experiments, subjects experienced a significant decrease in performance, a typical condition associated with excessive arousal that has probably affected other cognitive functions (like decision-making and selective attention). Furthermore, in both cases, frustration and temporal demand showed a significant increase after the stressful condition. 

The laboratory experiment was used not only to test the Neurometric in a controlled environment, but the data collected during this experiment were also used to perform a cross-task calibration of the machine learning model. Indeed, in a possible out-of-the-lab use of this technology, it would not be feasible to have calibration for the machine learning model coming from the same task the user will face (e.g., driving a car).

We observed that the proposed Neurometric can discriminate between low and high levels of stress during a multitasking activity with an AUC higher than 0.9 for resolutions higher than 40 s. Moreover, for resolutions lower than one second, its behaviour is not significantly different from both the SCL and machine learning approaches based on the Random Forest model. Observing the distribution obtained from the 18 participants, we found that even if the SCL does not significantly differ from the Neurometric and RF for a decimation higher than one second, its distribution shows higher dispersion than those obtained through the other methods. This result is likely due to the slower dynamics of the skin conductance tonic component (i.e., the SCL) with respect to EEG (Neurometric) and the derived parameters (RF). In fact, SCL usually varies with a time constant from 10 to a few tens of seconds, and therefore it becomes hard to discriminate between different conditions with higher time-resolutions. As a consequence (Figure 4 and Figure 7), discrimination performance represented by AUC values only tends to the Neurometric ones with lower time-resolutions, that is, greater than 40 s. This is a demonstration of the higher stability of the proposed Neurometric compared to SCL values. The results of the proposed Neurometric are competitive with other stress measurements for binary classification obtained using a limited number of EEG channels. Using just one EEG electrode to record brain activity in the beta band, the authors obtained 71.42% accuracy using an SVM classifier [66]. In the Ref. [67], the authors were able to discriminate between two levels of stress using three EEG electrodes and a KNN model with an accuracy of 99.26%. In the Ref. [68], the random forest model provided an accuracy of 78.6% in discriminating before and after stress conditions. 

As a further investigation of the similarities between the different proposed metrics in measuring stress, the trend of stress measures was analysed in terms of correlation and distance between the curves obtained for each subject. In fact, comparable results in terms of discrimination performance do not directly imply similarity between measures, as different discrimination performance (as in the comparison between EDA-based and EEG-related measures) can be due to completely uncorrelated measures or to correlated measures but with a different amount of noise. On the one hand, discrimination performances are related to the capability of specific measures to distinguish prolonged different states. On the other hand, high correlation would even include the possibility of following the temporal dynamics of the targeted state or phenomenon. In this case, the Neurometric trend is more similar, in terms of both correlation and distances, to RF-intra measures. The correlation between the two stress measures ranges from 0.85 for 10 s decimation to 0.87 for 30 s, and the RMSE does not overcome 0.2. The RF intra-stress measure shows medium correlation with SCL for 10 s resolution, increasing to 0.85 for 60 s. The distance between the curves is lower than 0.3. Comparable values of RMSE but lower values of correlation were found between Neurometric and SCL. Again, as previously discussed, the correlation of the EEG-related metrics with respect to SCL is undoubtedly due, especially with high time-resolutions (i.e., 10 s), to the intrinsic slow dynamics of the SCL itself. In fact, correlation (R) and similarity metrics (RMSE) tended to improve as the time resolution became lower (30 and 60 s). This outcome could also have been affected by the variability of SCL.

Based on this analysis during a multitasking session, we can conclude that the Neurometric is able to discriminate between two levels of stress and can provide the same performance, both from the discriminative and temporal point of view, as the RF model for which calibration is necessary. 

Therefore, the proposed Neurometric was tested during realistic driving. For this test, we decided to use both an intra-subject and a cross-task approach for calibration, where the multitasking data were used to train the model. For time resolutions higher than 10 s, the Neurometric provided high performance (AUC > 0.9) not significantly different from the SCL and RF-intra measures. However, the RF calibrated using the cross-task performed significantly worse than SCL, and for the Neurometric for 1 s decimation, significantly worse than the Neurometric for 10 s decimation. Moreover, the RF-cross distribution was more skewed than every other distribution, indicating the low stability of the stress measure computed through a cross-task approach. These results showed that cross-task calibration using the selected EEG features is not a viable alternative to the SCL, RF-intra or Neurometric measures. The analysis of the temporal trend of the Neurometric showed that even during realistic driving, there is a high correlation (R > 0.93) with the measures obtained by the RF-intra model and that the distance between the curves is lower than 0.19 in terms of RMSE. It must be noticed that the variability characterizing the RF-cross model is also reflected in the correlation and distances measures that are slightly worse than RF-intra. Finally, the correlation of the Neurometric with the SCL measure is medium (ranging from 0.48 to 0.60) and the distances between the curves vary from 0.27 to 0.29. 

The results of the proposed Neurometric are competitive compared to other stress measurements obtained during realistic driving. Even if in the literature, it is more common to find studies employing physiological features like SCL and heart activity-related features to assess the stress level of drivers [69], in the Ref. [58] the authors proposed an SVM model that, employing only EEG features, reached 97.95% of accuracy in discriminating rest and stress during driving, but not between stress levels. In this regard, it is worth highlighting that the EEG-based measure could be preferable for applications that need the assessment of mental and emotional states, the so-called passive Brain–Computer Interface applications [70,71], compared to measures coming from the autonomic system. Summarizing the evidence, measures like heart activity, ocular activity, and EDA showed a correlation with some mental states (e.g., stress, mental fatigue, drowsiness), but they were demonstrated to be useful and robust only in combination with other neuroimaging techniques directly linked to the central nervous system, that is, the brain [21,72,73]. Autonomic measures alone could be affected by external and not controllable factors, above all in a realistic setting. For example, SCL could vary not only because of the variation of stress, but due to the environmental temperature [74]. Pupillometry could highlight variations because of room luminance variations and not only because of workload variations, and so on [75]. On the contrary, using only brain signals may fail to take into account the multimodal aspect of stress, particularly that based on hormonal processes [21].

Some aspects could have influenced the obtained results like any other result in the literature: experiment duration, stressors administration, EEG processing, features extraction, and the type of classifier [34]. However, we would like to highlight that the proposed Neurometric is based on a straightforward computation, and on a well-validated approach used for EEG preprocessing in several contexts [15,76,77]. For this reason, this measure is suitable for realistic application, and it is less biased than a machine learning-based approach, where the calibration phase and model characteristics could unavoidably affect the results.

## 5. Conclusions

The proposed Neurometric was effectively tested during laboratory multitasking and validated during realistic driving. In both cases, it showed AUC higher than 0.9 in discriminating low and high levels of stress. Moreover, the proposed measure does not need calibration and can be used in real time both in and out of a laboratory to measure stress levels. Finally, the EEG employed in this experiment is characterized by a very simple setup. The low number of channels (i.e., two EEG sensors) used, the portable Bluetooth amplifier and gel-free electrodes suggest that this kind of technology is ready to be employed in out-of-the-lab applications.

## Figures and Tables

**Table 1:** Measures of dispersion of the AUC values obtained from the laboratory experiment. Values of interquartile range have been reported for each decimation and each variable.

Decimation (s)	SCL	Neurometric	RF-Intra
1	0.42	0.22	0.25
10	0.42	0.26	0.33
20	0.42	0.28	0.31
30	0.42	0.28	0.29
40	0.40	0.27	0.29
50	0.35	0.21	0.32
60	0.32	0.12	0.30

**Table 2:** Values of Pearson correlation (R) and the distance between curves computed in terms of Root mean squared error (RMSE) for the multitasking experiment. The median and interquartile range (IQR) have been reported.

		RF-Intra vs. Neurometric		RF-Intra vs. SCL		Neurometric vs. SCL	
Decimation (s)		Median	IQR	Median	IQR	Median	IQR
10	R	0.85	0.36	0.61	0.81	0.31	0.68
	RMSE	0.15	0.10	0.27	0.09	0.35	0.08
30	R	0.88	0.37	0.71	0.80	0.41	0.96
	RMSE	0.20	0.10	0.27	0.09	0.37	0.15
60	R	0.91	0.32	0.85	0.69	0.51	0.97
	RMSE	0.20	0.11	0.30	0.06	0.34	0.19

**Table 3:** Measures of dispersion of the AUC values obtained from the driving experiment. Values of interquartile range have been reported for each decimation and each variable.

Decimation (s)	SCL	Neurometric	RF-Intra	RF-Cross
1	0.10	0.11	0.11	0.33
10	0.10	0.04	0.06	0.33
20	0.09	0.03	0.04	0.29
30	0.08	0.02	0.03	0.25
40	0.07	0.02	0.02	0.21
50	0.06	0.02	0.02	0.19
60	0.05	0.02	0.02	0.16

**Table 4:** Values of Pearson correlation (R) and the distance between curves computed in terms of root mean squared error (RMSE) for the driving experiment. Median (Med) and interquartile range (IQR) have been reported.

		RF-Cross vs. Neurometric		RF-Cross vs. RF-Intra		RF-Cross vs. SCL		RF-Intra vs. Neurometric		RF-Intra vs. SCL		Neurometric vs. SCL	
Decimation (s)		Med	IQR	Med	IQR	Med	IQR	Med	IQR	Med	IQR	Med	IQR
10	R	0.87	0.20	0.81	0.32	0.43	0.42	0.93	0.04	0.61	0.30	0.49	0.33
	RMSE	0.22	0.11	0.27	0.11	0.30	0.07	0.19	0.06	0.28	0.14	0.29	0.08
30	R	0.91	0.19	0.81	0.29	0.48	0.46	0.96	0.05	0.68	0.35	0.51	0.42
	RMSE	0.24	0.14	0.25	0.13	0.28	0.08	0.11	0.07	0.25	0.10	0.27	0.09
60	R	0.92	0.15	0.83	0.37	0.62	0.30	0.96	0.05	0.72	0.23	0.61	0.54
	RMSE	0.28	0.12	0.22	0.12	0.26	0.13	0.12	0.11	0.23	0.06	0.29	0.09

## Data Availability

Data available on request due to privacy restrictions.
